# Everybody's Page

**Published:** 1893-02-11

**Authors:** 


### Everybody's Page.

A method, of French origin we believe, is suggested by which persons suffering from perspiring feet can be assisted. The remedy against this disagreeable condition consists of an absorbent dressing steeped in salicylic acid, worn between the foot and its covering of sock or stocking. It is stated that this obviates all that is objectionable.

At a meeting of the Darlaston School Board a protest against the employment of married women was raised, as being injurious to the home morally, and to the woman physically. In the face of this it might be interesting to know how many homes are not dependent on the labour of the mother of the family to save it from the workhouse. There are forms of labour unsuited to women under any circumstances, but it would be both difficult and even unfair to close all forms of livelihood against married women.

Those amongst us, who are equipped in well-versed argument against the social attractions of the girl of to-day are met by a formidable contradiction from the annals of the marriage rate itself, this rate pointing in 1891 to a higher figure than it has done for ten previous years. If we cannot thus measure the value of an article through its demand, we have yet to learn what test must be applied; but perhaps the expounders of this lamentable deterioration of the fair sex will suggest that the demand in this case anyhow has been in inverse ratio to its merits.

The Kangaroo has to thank the Westminster Aquarium for a good deal of notoriety. His tail, and its importance to its possessor as a means of support, is affording controversialists an object-lesson in the columns of a contemporary journal. It would appear that the tail of a Kangaroo must be regarded as a fifth limb—a fifth leg in fact, by authorities on the subject. We are referred to the Zoo for the proof of this opinion. The Boxing Kangaroo may be regarded as abnormal, so a visit to Westminster would not help on this occasion.

The Japanese method certainly robs dentistry of some of its terrors. Who is proof against the feeling of repugnance when the horrible collection of cruel-looking instruments is caught sight of when a visit to the dentist is necessary? In Japan a simpler method is resorted to by the dentist, who requires no instrument but his hands to extract the offending tooth. The necessary strength is obtained by a gradual strengthening of the fingers by special exercises, and the Japanese operator can accomplish his task with astonishing decision and rapidity.

England is not the only country by any means in which the professions are overstocked. Recent statistics show that in Naples there is a medical man to every 513 of the inhabitants. In a poor country like Italy it can be imagined on what wretched incomes many of the Neopolitan doctors have to exist.

Many an accepted idea is far from easy of complete comprehension. We often talk of eternity, whereas our minds cannot grasp the notion of half-a-million years even. Perpetual motion is another difficult matter to convey to our minds, but many years ago, in the "thirties," an American, by name John Randolph, very clearly elucidated the theory by taking for his object lesson the common things of everyday life, which he put thus neatly forward:—

Paper makes money,
Money makes banks,
Banks make poverty,
Poverty makes rags,
Rags make paper,
Paper makes money,
Money makes banks,

Once more Utah is free from the ban, and may now look forward to forming a recognised part of the American nation and becoming a flourishing State. The romance that attached to the existence of this peculiar people, it is true, will be gone, but the descendants of a race who, nothing daunted by hardship, poverty, and expatriotism, carried out the principles which their leaders had persuaded them into regarding as righteous, will not fail, we predict, to carry this inherent heroism into the ordinary channels of the recognised life of American citizens. We may therefore hope to hear good things of the Utah of the future.

That creation of the Continental brain, the "Long Distance" Ride, met its sequel last month in Vienna, where a number of ladies, not willing to be outdone by masculine feats, originated a scheme of their own, in the milder form of a "long distance" walk. About 40 ladies enrolled themselves on the list of competitors, the prize being carried off by a young married lady who completed the distance, some ten kilometres, in the quickest time. Perhaps this pedestrian feat, on the part of the fair sex, was milder in form than its masculine predecessor of equestrian fame; but the price of the winning, in this race of lesser glory, was not through the sufferings or the lives of dumb animals.

